# Perceived barriers, applied strategies, and typology of dentists treating patients with dental anxiety: a qualitative study

**DOI:** 10.1186/s12903-026-07886-7

**Published:** 2026-02-13

**Authors:** Philipp Klose, Daniel Lüdecke, Mats Mehrstedt, Daniel R Reissmann

**Affiliations:** 1 Department of Prosthetic Dentistry, University Medical Center Hamburg-Eppendorf, Hamburg, Germany; 2 Department of Medical Sociology, Center for Health Care Research, University Medical Center Hamburg-Eppendorf, Hamburg, Germany; 3 Dental Fears Clinic, Hamburg, Germany; 4 Department of Prosthodontics and Materials Science, University Medical Center Leipzig, Liebigstr. 12, Building 1, Leipzig, 04103 Germany

**Keywords:** Dental anxiety, Dentist typology, Patient–dentist relationship, Barriers in dental care, Qualitative research, Continuing education

## Abstract

**Background:**

Dental anxiety (DA) represents a significant challenge for patients and dental practitioners alike. While previous research has mainly focused on patients’ perspectives, less is known about how dentists themselves perceive and manage DA. This study aimed to explore interpersonal barriers, strategies, and experiences of dentists in the treatment of anxious patients and to develop a typology of dentists’ approaches.

**Methods:**

Semi-structured interviews with 60 dentists (15 specialized in DA treatment, 45 general practitioners) were conducted. The interview guide included 22 open-ended questions addressing definitions of DA, barriers, strategies, and personal experiences. Interviews were audio-recorded, transcribed verbatim, and analysed using qualitative content analysis. A typology of dentists was developed by clustering recurring response patterns across attitudes, strategies, emotional effects, and perceived helplessness.

**Results:**

Dentists recognized DA as a prevalent and clinically relevant problem that affects both patients and dental staff. Barriers included patient unreliability, limited financial reimbursement, time constraints, and lack of psychological support structures. Analysis revealed four distinct types of dentists: *Understanders*, *Demanders*, *Improvisers*, and *Unguided*. These groups differed systematically in their motivation, strategies, and openness to change. Successful concepts were often linked to continuing education or psychotherapeutic collaboration and were associated with reduced practitioner stress and improved patient–dentist relationships.

**Conclusions:**

This study suggests the presence of several interpersonal barriers in the management of DA and highlights substantial heterogeneity among dentists. The proposed typology offers a basis for tailored continuing education, curricular integration, and policy measures aimed at improving care for anxious patients while reducing practitioner burden.

**Supplementary Information:**

The online version contains supplementary material available at 10.1186/s12903-026-07886-7.

## Introduction

Despite improvements in dental equipment and pain control during dental treatment procedures, dental anxiety (DA) still remains a common and serious dental care issue for dentists and their patients [1–3]. Dental anxiety (DA) is a spectrum of fear-related responses to dental treatment, ranging from mild unease to severe phobia. In some cases, it may meet diagnostic criteria for specific phobia, with psychosomatic symptoms and significant impairment [4–6]. This abnormal fear or dread of visiting the dentist for preventive care or therapy and unwarranted anxiety over dental procedures can have a negative impact on oral health and is often associated with a low socioeconomic status [7, 8]. Causes of DA often seem to be either preceded by traumatic experiences, including adverse or abusive events in childhood, or transmitted through the social environment of fearful behaviour [9–12]. Epidemiological data suggest that approximately 3–5% of the general population suffer from a specific dental phobia as defined by diagnostic criteria. Around 10–15% report high dental anxiety with significant distress, while an additional 20–30% experience mild to moderate fear. Altogether, up to 50% may be affected by some form of dental anxiety [3, 7, 13, 14].

Considering the large proportion of subjects suffering from DA in the general population, every dentist is affected and, therefore, forced to deal with this issue. Treating anxious patients is often perceived as stressful for the dentist [15]. Some reasons are appointment cancellations, delays in appointment schedule, longer time spent on treatments, and ineffective or counterproductive treatment approaches. Dentists experienced increased stress when anxious patients made complaints they deemed unwarranted [16]. As a result, dentists often avoid treatment of patients with DA, a phenomenon reported in previous studies linking anxiety management to increased stress and perceived burden among practitioners [15, 17]. From an ethical perspective, this avoidance raises concerns regarding equal access to dental care and professional responsibility. Patients with dental anxiety represent a vulnerable group, and systematically avoiding their treatment could conflict with the ethical principle of justice and the dentist’s duty of care. The denial of necessary dental treatments can cause a considerable deterioration of the oral health status, which in turn can lead to guilt, shame, depression, social isolation and decreased quality of life [18–20]. The consequences of dental anxiety do not only affect the patients but also the environment of the patients, the patient care in dental practices and the entire practice team [17, 21–23].

Although the origins of dental anxiety often lie outside the dental setting, dentists still play an important role in the emergence and maintenance of DA. It is therefore essential to investigate how dentists perceive and evaluate their working relationship with anxious patients. Furthermore, dentists also play a central role in the avoidance and reduction of dental anxiety [24]. Surprisingly, dentists’ views of treating fearful patients are not well described so far [5, 15, 17, 21–23]. Many dentists feel inadequately prepared for patients with DA [17, 22, 25–27]. Studies showed that many dentists would appreciate treatment approaches to maximize the effectiveness of dental fear interventions for the patients [24]. Furthermore, since most of the studies dealing with the dentist’s barriers of treating patients with DA are somewhat outdated [22, 28] and a lack of applicable instruments measuring these barriers was evident, a qualitative approach with an inductive procedure to achieve an optimum adaptable interview guide seems to be necessary [29].

This study aimed to identify interpersonal barriers between dentists and patients with dental anxiety and to examine how dentists’ perceptions, approaches, and strategies vary across a diverse group of practitioners. Based on response patterns, the study sought to develop a typology of dentists with respect to dental care for anxious patients.

## Methods

### Study design

A qualitative research design was employed in this study to explore subjective experiences and identify patterns within semi-structured interview data. The data were analysed using qualitative content analysis following Mayring’s approach [30], which provides a systematic framework for both inductive and deductive category development. To deepen the interpretive dimension and enable theory generation beyond pre-structured themes, selected elements from Grounded Theory methodology were integrated [31]. This combination offers a structured yet flexible approach to understanding the underlying patterns in complex qualitative data.

### Sample and data collection

In this qualitative study, 60 dentists were selected as convenience sample in both rural and urban parts in Germany. The selection criteria focused on dentists that either had widely recognized reputation for the treatment of patients with dental anxiety or had announced special qualifications after further training in this area in their external advertising. These individuals were identified through professional networks, continuing education programs, and publicly available practice information indicating experience with anxious patients. The inclusion of both specialized and non-specialized dentists was deliberate to capture diverse perspectives. As the findings show, even highly experienced dentists differ considerably in their attitudes and coping strategies, making the emergence of negative or unstructured profiles neither unexpected nor contradictory.

We used a comprehensive semi-structured interview guide to explore dentists’ experiences, attitudes, and practices in dealing with dental anxiety. This approach allowed for the collection of both structured and nuanced data across multiple dimensions, providing the empirical basis for systematic content analysis and typology development. The interview guide was developed specifically for this study, and an English-language version is available as a supplementary file (Appendix 1). In addition to exploring perceived barriers and strategies, the interviews were designed to capture recurring behavioural and attitudinal patterns among dentists, which later led to the development of the typology.

Before final data collection, pilot interviews were conducted with 15 individuals. Based on the pilot study, the interview guide was minimally adjusted to enhance clarity and focus. Specifically, wording of two questions addressing coping strategies was simplified, and an additional prompt about dentists’ emotional responses was added. The sample was expanded to 60 participants to achieve a broad range of professional backgrounds, ages and genders and to capture a wide variety of relevant perspectives [32]. We refer to saturation not in terms of participant numbers, but as analytical saturation—meaning the absence of new codes or themes emerging during the iterative process of analysis [33–36].

All interviews were conducted in German by the same trained dentist (PK) in the participants’ practice settings, ensuring consistent conditions and eliminating the need for calibration. Participation was voluntary and respondents did not receive any compensation which was communicated in advance. All interviews were audio-taped and transcribed verbatim. The interviews lasted between 40 and 70 min, with a mean duration of approximately 50 min, allowing sufficient depth and exploration of the interview topics.

The interview guide for the semi-structured interviews consisted of 22 open-ended questions in four sections:

(i) Reported barriers, definition and gradation of dental fear.
(ii) Approach to anxious patients, handling of major barriers and impact on the dentist.
(iii) Proposed solutions and incentives.
(iv) Methods applied and state of knowledge.

### Data analysis

All transcripts were checked for completeness and anonymised prior to analysis. The data were imported into MAXQDA© 11 plus (Verbi Software, Berlin), a software program for managing qualitative data. The analysis followed the principles of qualitative content analysis as described by Mayring, complemented by elements of Grounded Theory to deepen theoretical interpretation [30, 31].

First, all transcripts were read repeatedly to achieve familiarisation with the material. During open coding, meaningful segments were identified and labelled inductively without applying predefined categories. Through constant comparison within and across interviews, codes were progressively grouped into higher-order categories. In a subsequent step, axial coding was used to explore how categories related to one another, for example in terms of links between perceived barriers, emotional responses, and coping strategies. Analytical memos were written throughout the process to document interpretive decisions and enhance transparency.

The typology was developed through a cross-case analysis in which recurring patterns across four dimensions—attitudes, approaches, emotional effects, and perceived helplessness—were compared and clustered into conceptually coherent groups. This iterative and systematic procedure ensured transparency and traceability. Representative quotations were selected to illustrate the categories and to demonstrate how interpretations emerged directly from the data [29, 37].

### Definition of subcategories and response clusters

To further analyse the data, we followed an adapted Grounded Theory approach [31], starting with open coding to break down participant responses into discrete concepts. While initial codes were partly informed by predefined themes derived from the interview guide, the coding process remained open to inductively emerging concepts.

For instance, a dentist’s statement, *“Yes*,* there are moments when I feel utterly helpless because I don’t have the right words or techniques to soothe their overwhelming fear*,*”* was assigned codes such as *“Feeling helpless*,*” “Lack of effective communication*,*”* and *“Inadequate coping strategies.”*

Next, we used axial coding to group these initial codes into broader categories that revealed deeper connections. The codes *“Feeling helpless”* and *“Emotional impact on the dentist”* were grouped under the category *“Emotional Response of Dentists.”* Similarly, *“Lack of effective communication”* and *“Inadequate coping strategies”* formed the category *“Perceived Professional Challenges.”*

This systematic and iterative approach enabled us to move from granular data to a more comprehensive understanding of the core themes—illustrating, for example, how overwhelming patient anxiety contributes to dentists’ feelings of helplessness due to a perceived lack of effective coping strategies.

### Reflexivity and researcher positioning

The interviews in this study were conducted by a trained dentist (PK) with clinical experience in treating patients with dental anxiety. This professional background facilitated rapport with participants but also required continuous reflection to minimize interpretation bias. To enhance reflexivity, the researcher kept analytical memos during coding and repeatedly revisited interpretations together with the other authors (DL, MM, DR), who brought methodological expertise and external perspectives to the analysis. This collaborative and iterative approach aimed to ensure that the findings reflected the participants’ accounts rather than the researcher’s preconceptions [38].

### Typology development

As the structured analysis progressed, distinct response patterns emerged, indicating systematically recurring patterns of perceptions, coping strategies and professional attitudes. These patterns were differentiated along four thematic dimensions: Attitude, Approach, Effects, and Helplessness. Based on these dimensions, a typology of dentists was developed. Group affiliation appeared to be further influenced by contextual factors such as personality traits, prior knowledge, and professional experience.

The typology was derived from the structured content analysis and based on response patterns observed in the defined dimensions. To ensure its validity, the classification was carried out systematically based on illustrative responses. The distinguishing features of each type were compared in a summary table, and reflective memos as well as analytical logs were revisited to support and document the decision-making process [30, 37].

## Results

### Participants’ characteristics

The 60 participants included general dentists (*n* = 37), pediatric dentists (*n* = 3), oral surgeons (*n* = 5) and dentists specialized in patients with DA (*n* = 15) with a balanced distribution in age and gender (Table 1). Most participants worked in private dental practices (*n* = 54), while the remainder were employed in public or university-affiliated settings (*n* = 6).

**Table 1:** Participants’ characteristics

Category		Total (*n* = 60)	Gender		Age group	
			Men (*n* = 31)	Women (*n* = 29)	≤ 35 years (*n* = 23)	> 35 years (*n* = 37)
		*n (%)*				
Localization						
	Urban	36 (60.0)	20 (64.5)	16 (55.2)	14 (60.9)	22 (59.5)
	Small town	16 (26.7)	8 (25.8)	8 (27.6)	5 (21.7)	11 (29.7)
	Rural	8 (13.3)	3 (9.7)	5 (17.2)	4 (17.4)	4 (10.8)
Specialization						
	Yes	15 (25.0)	7 (22.6)	8 (27.6)	2 (8.7)	13 (35.1)
	No	45 (75.0)	24 (77.4)	21 (72.4)	21 (91.3)	24 (64.9)

### Perceived barriers

Participants described dental anxiety—particularly fear of pain—as one of the most persistent challenges in everyday dental care. The interviews revealed several recurring barriers, including financial constraints related to insurance regulations, difficulties in patient reliability, and unrealistic expectations regarding treatment outcomes. Participants also highlighted limited institutional structures for psychological support, which complicated the management of highly anxious individuals. Although fewer participants commented on structural or legal obstacles, these were still mentioned as potential sources of uncertainty in complex cases.

Interview accounts emphasized that dental fear influences clinical interaction across various disciplines rather than being limited to specific procedures. As one participant stated, ‘Even when patients know they need treatment, fear often wins over reason. They cancel or simply don’t show up.’ (Participant #17, general dentist). Another explained that small deviations in patient behaviour ‘can already hint at DA and affect everything from communication to prophylaxis’ (Participant #53, specialist in dental fear treatment). These accounts illustrate how dental anxiety affects interpersonal dynamics as well as practical aspects of treatment planning.”

### Definition and gradation of dental fear

Participants described dental anxiety as a multidimensional phenomenon ranging from mild unease to pronounced fear with psychosomatic symptoms. They reported that patients express dental fear in various ways, including physical reactions such as sweating, tremors, hyperventilation or circulatory discomfort, and behavioural responses such as avoidance, missed appointments, or seeking emergency-only care.

Several dentists highlighted the importance of recognising different levels of severity, noting that highly anxious individuals often appear only in acute or emergency situations, whereas patients with mild or moderate fear are more commonly encountered during routine care. One participant explained: ‘Only the tip of the iceberg actually appears in regular practice. Many of the truly anxious patients show up only in emergencies or never come at all.’ (Participant #52, general dentist).”

### Approach to anxious patients

Across interviews, dentists described adapting their clinical approach when treating anxious patients, most commonly by beginning with non-invasive conversations and gradually introducing diagnostic or therapeutic steps. Many emphasized the importance of relationship-building measures such as clear explanations, discussing fears openly, or demonstrating instruments before use. A DA-specialised dentist explained, ‘I always start with a talk, not a treatment. When patients feel heard, half the fear is already gone.’ (Participant #42, DA-specialized dentist). Several dentists described intentionally spacing initial contact, diagnostics and treatment across multiple appointments to give patients time to adjust. One general dentist noted, ‘I talk to patients about their fears, trying to understand the cause. If they can become familiar with me and the environment step by step, treatment becomes much more manageable for both sides.’ (Participant #39). Participants described a wide spectrum of attitudes toward treating anxious individuals. Some expressed discomfort or emotional strain, whereas others reported neutral or even positive experiences, particularly when trust-building strategies had proven successful. Difficulties such as inconsistent attendance, unclear treatment goals or emotional overload were mentioned as factors that occasionally limited success.

### Major problems

Participants described several recurring challenges when treating anxious patients. A frequently mentioned theme was the difficulty of relying on patients with heightened fear—many struggled with keeping appointments, tolerating longer procedures, or maintaining consistent motivation. Dentists also reported that managing DA often required substantially more time and emotional energy, which they felt was not adequately compensated within current reimbursement structures.

Another issue described across interviews concerned uncertainty about psychological management. Several dentists expressed insecurity about whether their communication style or chosen approach was truly helpful in acute fear situations. One general dentist noted: ‘Everything takes longer, and I sometimes feel I don’t have the right lever to really reach these patients.’ (Participant #40). Another explained: ‘I often don’t even know if I’m doing it right psychologically. Maybe I should be more confident—but I’m afraid of doing something wrong.’ (Participant #43, general dentist). These accounts highlight how emotional demands, time constraints and structural limitations jointly contribute to the perceived burden of managing anxious patients.

### Impact on the dentist

Across interviews, dentists described the emotional and psychological demands of treating anxious patients as particularly challenging. Accounts emphasised that managing fear-related behaviour required sustained concentration, flexible communication, and continuous reassurance, which some dentists likened to ‘a form of high-performance work’. One oral surgeon noted: ‘You need mental endurance—sometimes more than technical skill.’ (Participant #8).

Several participants described feeling exhausted after such appointments, especially when managing cases with pronounced fear or additional psychological difficulties. As one dentist expressed: ‘I am whacked after these appointments — mentally, sometimes even physically.’ (Participant #07). Others similarly reported moments of insecurity or temporary helplessness, particularly when their usual strategies proved insufficient. One clinician reflected: ‘It can be draining when nothing seems to work—you start questioning whether you are doing the right thing.’ (Participant #27). These narratives illustrate how emotional strain, uncertainty and increased cognitive load shape dentists’ experiences when working with anxious patients.

### Proposed solutions and incentives

Participants described a variety of ways in which the management of anxious patients could be improved. A recurring theme across interviews was the need for more time and appropriate financial compensation to reflect the additional communicative and emotional effort required. Several accounts also pointed to a lack of accessible psychological support structures, leading dentists to request closer collaboration with psychotherapists or specialised clinics.

Participants highlighted the importance of early preventive interventions and improved dental education. One specialist commented: ‘You should prophylactically prevent that people get scared. In Sweden, for example, prophylaxis is strongly supported—and coaching the team could be helpful.’ (Participant #16). Others emphasised the value of structured referral pathways: ‘Earlier referrals to specialised colleagues, such as surgeons or pediatric dentists, can make treatment much more manageable for everyone involved.’ (Participant #12).

Participants stressed the relevance of better undergraduate and postgraduate training in managing anxious patients, noting that practical, experience-based approaches would be particularly beneficial. As one general dentist explained: ‘It all depends often on the training.’ (Participant #40). These perspectives underline the need for systemic adjustments in reimbursement, interdisciplinary cooperation and professional education to support more effective care for anxious patients.

### Methods applied and state of knowledge

Participants described a wide range of methods used to manage dental anxiety. Several accounts highlighted the use of communicative and behavioural strategies, such as explaining each step of the procedure, allowing patients to signal breaks, or using distraction through conversation. Some dentists also reported applying relaxation techniques or elements of graded exposure, although many emphasised that they had acquired these skills informally rather than through structured training.

Pharmacological interventions such as oral sedation or nitrous oxide were used by some, particularly in cases of pronounced fear or when communicative techniques were insufficient. However, several participants expressed uncertainty about whether these interventions addressed the underlying anxiety or merely enabled short-term treatment. One clinician noted: ‘Sedation can help in the moment, but it doesn’t solve the fear. You’re only treating the episode, not the cause.’ (Participant #31).

Knowledge about psychological concepts related to dental fear varied considerably. While some dentists felt familiar with cognitive or behavioural principles, others expressed limited confidence and described relying largely on personal experience. As one dentist explained: ‘I learned most of this by trial and error — not in university.’ (Participant #22). These narratives suggest that methods differ widely and that structured education may help to standardise and support anxiety-related care.

For more details on the topics above, see Appendix 2.

### Typology of dentists

Based on recurring patterns across the interviews, four qualitatively distinct profiles of dentists emerged. These types differed in their attitudes toward anxious patients, their preferred strategies, and their emotional responses during treatment. The typology does not reflect numerical distributions but rather conceptual clusters illustrating the range of approaches identified in the material (Table 2).

**Table 2:** Characteristics of types

Type	Attitude	Approach	Education	Helplessness
Type I – *Understanders*	Positive / empathetic	Active measures (e.g., communication, sedation)	Already engaged	Rarely helpless
Type II – *Demanders*	Neutral / reflective	Behavioural strategies	Interested, but critical of offers	Low
Type III – *Improvisers*	Dissatisfied / stressed	Improvisation	Limited	Occasionally
Type IV – *Unguided*	Sceptical / negative	No structured approach	Rejected as impractical	High

#### Type I: the Understanding type with minor weaknesses – understanders

These dentists approached anxious patients with empathy, patience and a strong focus on relationship-building. They viewed fear as a legitimate and clinically relevant factor and described taking time to explain procedures, offer reassurance and adjust the pace of treatment. One participant stated: ‘I always want to know what exactly is worrying the patient — only then can we take the next step together.’ (Participant #42). For these clinicians, successful treatment was closely tied to trust and communication.

#### Type II: the demanding type with self-doubt – demanders

This group emphasised efficiency, structure and technical aspects of treatment. Anxious behaviour was often perceived as disruptive or delaying, and some dentists expressed frustration when fear interfered with workflow. A dentist explained: ‘If the patient cannot keep up with the procedure, it becomes difficult to deliver what needs to be done.’ (Participant #12). Although they aimed to complete treatment effectively, their strategies tended to be more directive and less focused on emotional support.

#### Type III: the dissatisfied improvisers – improvisers

Improvisers described feeling uncertain or underprepared when dealing with anxious patients. Their approaches varied widely from case to case, often depending on intuition or situational judgment rather than structured techniques. One dentist noted: ‘I just try something different each time — I’m never quite sure what really helps.’ (Participant #27). This type reflects clinicians who are motivated to help but lack consistent strategies, training or confidence.

#### Type IV: the practitioners without guidance – unguided

These dentists reported experiencing the management of anxious patients as emotionally taxing and at times overwhelming. They described feelings of helplessness, insecurity and limited control, especially when fear escalated unexpectedly. A participant expressed: ‘Sometimes I feel lost — whatever I try, it doesn’t seem to work.’ (Participant #07). This group highlighted the emotional burden and the need for clearer guidance, training and structural support.

For more details on the typology of dentists, see Appendix 3.

## Discussion

To our knowledge, this is the first qualitative study to systematically explore dentists’ perspectives, strategies, and perceived barriers in treating patients with dental anxiety (DA), while also identifying typological patterns in their professional attitudes and behaviour. Findings confirm that DA remains a persistent issue for both patients and practitioners, with diverse barriers affecting treatment dynamics and the wellbeing of dental staff. However, dentists showed substantial variability in how they perceived and addressed these challenges.

This study identified key interpersonal barriers and coping strategies in the treatment of patients with dental anxiety. Our findings align with earlier studies confirming the persistent presence and burden of DA in dental practice [39–41]. Especially among younger and less experienced professionals, DA was more frequently described as a clinical challenge. While much of the literature has focused on patients’ perspectives, few studies have explored how DA affects dentists themselves [1, 21].

Dentists reported persistent challenges such as patient unreliability, emotional strain, and limited institutional support, consistent with previous research highlighting communication difficulties and stress in treating anxious patients [15, 17, 21, 26]. Our findings further confirm that effective management is closely tied to dentists’ self-efficacy, access to training, and structural conditions such as time and reimbursement constraints. Literature also emphasizes that dentists often feel underprepared or unsupported when treating anxious patients [16, 26, 27]. Consistent with these reports, our study identified a multifaceted set of barriers: psychological strain, lack of structured approaches, unclear treatment goals, financial constraints, and insufficient access to psychological support or collaborative models. Some participants experienced ambivalence between their professional values and the emotional demands of DA treatment, contributing to feelings of inadequacy or burnout [21]. In Germany, dental care for adults is primarily financed through the statutory health insurance system, which covers basic treatments but often requires partial out-of-pocket payments for more complex or extended procedures. Evidence shows that these co-payment structures and the mixed financing system influence patient decision-making and shape how dental care is organized [42, 43]. Such structural constraints may indirectly increase the burden experienced by dentists when providing anxiety-sensitive and time-intensive care.

Beyond these general patterns, the analysis revealed systematic variations in how dentists perceive and handle anxious patients, which were conceptualized in a typology of four characteristic groups. Although typological approaches have been applied in other healthcare professions to tailor educational interventions (e.g., nursing, psychology), few attempts have been made to classify dentists according to their communication styles or emotional coping mechanisms [27, 41]. In our study, factors such as professional experience, age, and educational background appeared to influence group affiliation. The identified types displayed internal consistency in their characteristics, while differing significantly from each other. Most participants recognized the prevalence and clinical relevance of DA. However, attitudes toward DA varied: *Understanders* and *Demanders* expressed more positive or neutral views, whereas *Improvisers* and *Unguided* types tended toward negative or indifferent attitudes. Only a few dentists appeared entirely content with their current approach and disinterested in further improvement.

*Improvisers* and *Unguided* dentists reported the greatest emotional burden and the least structured strategies. Yet, a key distinction emerged: *Improvisers* showed reflective capacity and openness to change, suggesting they may benefit from targeted interventions that enhance communication skills and reduce situational stress. *Unguided* dentists, in contrast, often displayed resignation and a fixed mindset. Supporting this group may require long-term mentoring structures, institutional guidance, and possibly phased exposure approaches.

*Understanders* and *Demanders*, in contrast, frequently shared success stories and perceived reductions in patient fear over time, particularly through trust-building and strategic communication. These groups may serve as role models within the profession, as their patient-centered mindset, training background, and accumulated experiences often led to a self-reinforcing cycle of success. Interestingly, the perception of barriers differed between groups: while negatively oriented dentists emphasized stress, insecurity, time pressure, and inadequate reimbursement, more positive respondents highlighted loyal patient relationships, satisfaction with outcomes, and successful fear-reduction experiences.

These findings align with international research describing similar challenges in the management of anxious patients. Studies from the UK and Scandinavia have reported that dentists often feel insufficiently prepared for communication-intensive encounters and experience increased psychological demands when treating fearful individuals [44, 45]. Comparable work from Australia emphasizes the importance of structured training in behavioural techniques and early anxiety prevention [46]. Our typology extends this literature by illustrating how varying levels of confidence, emotional responses and coping strategies shape clinical behaviour across different professional contexts.

A major strength of this study lies in the breadth of experiences and the diversity of professional backgrounds represented in the sample, which provided rich and varied insights into the management of dental anxiety. Moreover, this study integrates structural, emotional, and procedural dimensions, offering a comprehensive and practice-relevant perspective. Limitations include the single-researcher analysis, which may have introduced interpretative bias despite rigorous coding protocols. Furthermore, the professional background of the interviewer as a fellow dentist may have influenced responses through social desirability. Nevertheless, consistent analytic procedures and the use of reflective memos supported transparency and reliability in interpretation. Although the sample included dentists with recognized expertise in the management of anxious patients, the emergence of less structured or more burdened profiles is not contradictory. Even highly experienced clinicians differed substantially in their attitudes, confidence and coping strategies, and the purposive sampling intentionally allowed for this variation. The typology therefore reflects the diversity of approaches within everyday practice rather than a gradient of expertise.

The typology developed in this study holds clinical and educational value. A differentiated approach to training—aligned with dentists’ attitudes, needs, and motivational levels—may improve the effectiveness of DA interventions. For example:

### Clinical level

*Improvisers* may benefit from short, success-oriented communication tools. *Unguided* types require long-term mentoring and attitudinal change support. To apply the typology in clinical and educational practice, future research is needed to develop a brief self-assessment questionnaire to determine dentists’ profile. Such tools could be integrated into continuing education or used as reflective instruments in postgraduate training, helping practitioners recognizing personal strengths and challenges in managing dental anxiety.

### Educational level

Undergraduate and postgraduate curricula should include structured training on DA, integrating theoretical foundations and practical simulations. Typology-informed modules could enhance learner engagement.

### Policy level

Health systems should develop referral networks, improve reimbursement for complex DA cases, and promote interdisciplinary cooperation (e.g., with psychologists).

Participants frequently called for practical, evidence-based training programs, including cognitive-behavioural methods, relaxation techniques, and desensitization strategies. Promoting structured and reimbursed care models could lead to mutual benefits—improving patient experience while reducing practitioner strain [15]. This typology-based approach allows for tailored interventions and avoids the limitations of one-size-fits-all strategies. Tailored educational and policy strategies—potentially tested through pilot training programs—could be refined based on feedback and long-term evaluation. Future research may also explore digital innovations such as virtual reality in DA management [47]. These findings are consistent with prior work showing that communication-focused interventions can significantly reduce dentists’ stress and improve patient cooperation [15, 24]. Incorporating typology-informed reflection into training curricula may foster more adaptive strategies and empathy, ultimately enhancing treatment outcomes and practitioner well-being.

## Conclusion

Dental fear remains a major challenge in outpatient care and is associated with the persistence of interpersonal barriers—such as patient unreliability, emotional strain, and structural limitations—that continue to challenge effective care. Addressing these barriers through communication-focused and system-level interventions remains essential for improving patient outcomes and practitioner well-being. Since dentists differ widely in their ability to manage affected patients—from well-trained professionals with established strategies to practitioners lacking guidance, effective solutions will therefore require approaches tailored to the needs of different dentist types. Recognizing these profiles may guide the design of targeted training and individualized support.

## Supplementary Information

Supplementary Material 1.

Supplementary Material 2.

Supplementary Material 3.

## Data Availability

The datasets generated and analysed during the current study are available from the corresponding author on reasonable request.
