# Supplementary material for: Perceived barriers, applied strategies, and typology of dentists treating patients with dental anxiety: a qualitative study
Source: BMC Oral Health. 2026 Feb 13;26:422. doi: 10.1186/s12903-026-07886-7 (PMC12955291; doi:10.1186/s12903-026-07886-7)
Supplement: Supplementary file 1 — Supplementary Material 1. [file 12903_2026_7886_MOESM1_ESM.docx]

Question 1: In your opinion, what are the most difficult aspects of dental treatment today?

Question 2: How would you define dental anxiety?

Question 3: What percentage of your patients would you say have…
- severe dental anxiety (DA) ___
- mild DA ___
- no DA ___

Question 4: Does your approach to treatment differ from the usual when you know a patient suffers from DA? If so, in what way?

Question 5: Do you enjoy treating patients with DA? Why or why not?

Question 6: Are you satisfied with the treatment outcomes of these patients? Please explain.

Question 7: Please name the two biggest problems you encounter when treating patients with DA in your practice.

Question 8: What would need to happen in order to overcome these problems?

Question 9: Would you like to be able to help these patients more effectively?

Question 10: What effects do you notice on yourself when treating patients with DA?

Question 11: Are you aware of methods to reduce patients’ anxiety? Do you have experience with them? Have you considered learning them? (e.g., general anesthesia, hypnosis, sedatives)

Question 12: Do you feel that patients’ DA decreases during treatment with you? How does this manifest itself?

Question 13: Do you sometimes feel helpless when treating patients with DA? If yes, in what way?

Question 14: Should the treatment of patients with DA generally be the responsibility of specialists and university hospitals?

Question 15: Who, besides the patients themselves, would benefit if patients with DA could be treated more effectively?

Question 16: Would you say that patients with DA place special demands on the skills of the dentist? …on the overall treatment? Which ones?

Question 17: Would you say that, at your current stage, you are able to treat patients with DA adequately?

Question 18: In your opinion, should undergraduate dental training be improved with regard to treating patients with DA?

Question 19: What do you think about postgraduate training opportunities in this area? Do you have experience with them?

Addon: Is there anything else you would like to tell me about anxious patients?

Question 20: Now I would like to ask you about pain patients who come to your practice more or less without an appointment. Please think back to the past week: How do you usually treat these patients?

Question 21: Were you usually able to carry out the treatment you had planned?

Question 22: Do these patients pose any particular problems?
