# Supplementary material for: Perceived barriers, applied strategies, and typology of dentists treating patients with dental anxiety: a qualitative study
Source: BMC Oral Health. 2026 Feb 13;26:422. doi: 10.1186/s12903-026-07886-7 (PMC12955291; doi:10.1186/s12903-026-07886-7)
Supplement: Supplementary file 2 — Supplementary Material 2. [file 12903_2026_7886_MOESM2_ESM.docx]

## Appendix 2 – Additional Qualitative Results

The following supplementary material presents additional findings from the qualitative content analysis that could not be included in the main manuscript due to space limitations and the need for thematic focus. These results provide further differentiation of identified categories and subtypes, supported by illustrative quotes that deepen the understanding of the reported phenomena.

**Perceived barriers**

Participants consistently identified dental anxiety as one of the most persistent challenges in dentistry. One third of respondents highlighted fear of pain and stigmatization of fearful patients as major barriers. In addition, financial issues—particularly in relation to insurance-covered treatments—were frequently mentioned. Poor compliance or unrealistic expectations of treatment outcomes were cited by roughly 40% of participants.

Professional barriers, such as surgical or orthodontic procedures, pediatric treatment, or legal and guideline-related constraints, were less commonly reported. With regard to fear as a barrier, several responses were similar in substance:

„Nevertheless, little abnormalities in patient management or differences from everyday situations can still be related to DA or point it out. Fear of dentistry as an entire area also leads inevitably to abnormalities in the various disciplines such as prophylaxis.“ (ID 53, specialist in dental fear treatment)

**Definition and gradation of dental fear**

On average, participants estimated that 15% of their patients had severe dental anxiety, 50% moderate fear, and 35% little to no fear. When asked how they defined “dental fear,” respondents described a wide range of symptoms: cold sweat, circulatory problems, tremors, hyperventilation, lethargy, delusions, physical tension, vigilance, as well as behavioural patterns such as non-compliance or “practice hopping.”

Many emphasized the importance of distinguishing between levels of severity, with most patients belonging to the mild or moderate group. One dentist explained:

"[…] only the tip of the iceberg. The majority of these patients appears actually only in emergency service or appears never.“ (ID 52, general dentist)

**Approach to anxious patients**

Most participants stressed that additional communication and a calm, stepwise approach are essential for successful treatment of anxious patients. About one third reported that they deliberately spread initial contact, diagnostics, and first treatment across several appointments. Some routinely asked patients about dental fear, while a minority treated all patients in the same way, regardless of anxiety status.

Examples of approaches included:

„At the beginning the patient notes his anxiety on the medical history form. A relationship of trust must be created; first we start talking only about professional, non-dental issues. The treatment is introduced by detailed explanations and a lot of demonstrations on our part. Patients are being warned before single steps; Anesthesia always starts with topical anesthesia. Also, the anesthesia is injected very slowly; interestingly, these patients always want anesthesia and are often patients with severe pain; further, we constantly give a temporal feedback how long a treatment step will last.“ (ID 07, general dentist)

„I talk to the patients about their fears, trying to figure out the cause. Thus you can often already find an access to the patient. More difficult, expensive or protracted actions I move far back in treatment time to give the patient time to get used to me. Once they have gotten their courage up, a benefit is achieved for both parties - me and the patient.“ (ID 39, specialist in dental fear treatment)

Attitudes toward treating anxious patients varied: one third disliked it, one third were indifferent, and one third even enjoyed treating these patients. Illustrative statements included:

„I don’t like to treat patients with severe anxiety, because I am often the last address. The pressure is greater than usual.“ (ID 08, pediatric dentist)

„Yes, [I do like to treat these patients] very much. Our most loyal and satisfied patients describe themselves as former anxiety patients.” (ID 05, general dentist)

While most were satisfied with treatment outcomes, some expressed frustration, often due to unclear treatment goals, missed appointments, or reliance on emergency visits:

„I do my best - you always have to keep the time in mind and not all the patients accept, what is medically recommendable.“ (ID 27, oral surgeon)

**Major problems**

When asked to name the two most important problems related to dental fear, respondents highlighted unreliability of patients and the disproportionate effort required compared to reimbursement. Additional problems included poor oral hygiene, limited treatment tolerance, and heightened patient expectations.

Examples included:

„Everything takes longer and therefore it is not worth the money. Often it simply lacks the training and I cannot find the right lever to be able to mentally grasp the patient.” (ID 40, general dentist)

„I often do not even know if I'm doing it right with psychology. Maybe I should approach the patient more confident - on the other hand, I'm afraid that I'm doing something wrong.“ (ID 43, general dentist)

**Impact on the dentist**

Most dentists described the emotional and psychological aspects of treating anxious patients as the main challenge, while technical or logistical barriers were less prominent. Roughly half reported that such treatments were more stressful and required greater concentration:

„I explain a lot more. It is very challenging, comparable to high-performance sports.“ (ID 05, general dentist)

„Stressed, wearying, especially [when treating] a stressful child. I am whacked, mentally, sometimes annoyed.“ (ID 07, general dentist)

Others emphasized that experience and routine helped reduce stress:

„There is a good feeling on success. During the treatment itself I am also excited - it has a lot to do with experience.“ (ID 16, specialist in dental fear treatment)

Although many felt reasonably capable of managing anxious patients, about half admitted occasionally feeling helpless—particularly with patients who had comorbid mental health issues. Descriptions of helplessness included compromised treatment outcomes, referrals for surgery or general anesthesia, or seeking assistance from colleagues.

Respondents also emphasized the broader impact of improving care for anxious patients, pointing out benefits for health insurance systems, practice teams, and families:

„Early prophylaxis prevents costly treatments, which would lead to a relief of the health insurance, [and also] for the practice team: stress-free work would be possible.“ (ID 01, general dentist)

„Your children, the fear is being transferred from the parents to their children. Either the children are of tiptop oral hygiene or comparably traumatized." (ID 10, specialist in dental fear treatment)

**Proposed solutions and incentives**

Most participants emphasized the need for more time and adequate financial compensation for treating anxious patients. Additional demands included easier access to psychotherapeutic support, earlier preventive interventions, and improved dental education. Less frequently, respondents suggested enforced patient-dentist contact, technical solutions (e.g., nitrous oxide, general anesthesia), or referral to specialized colleagues.

Examples included:
"…earlier referrals to specialized colleagues, such as surgeons or pediatric dentists" (ID 12, general practitioner)
“You should prophylactically prevent that people get scared. In Sweden, for example, prophylaxis is strongly supported and coaching the team could be helpful.” (ID 16, specialist in dental fear treatment)
"Better education and thereby faster and better treatment. It all depends often on the training." (ID 40, general dentist)

**Methods applied and state of knowledge**

Many dentists reported offering specific methods to enable treatment, such as general anesthesia (one third), sedatives (one quarter), or nitrous oxide. Communication techniques and, less frequently, hypnosis were also mentioned, alongside alternative approaches such as behavioral therapy, NLP, kinesiology, homeopathy, or Bach flower remedies.

Most participants observed that fear tends to diminish over the course of treatment:

"Yes, absolutely [fear decreases], at 99%, we have experienced so far. Trust is the key to freedom from fear. If we create trust, the patient loses the fear." (ID 44, general dentist)

However, some restricted this to patients with moderate anxiety:

"Treatment in patients with moderate anxiety: yes. Concerning extremely anxious patients: it is a success to get them to come back." (ID 34, general dentist)

Specialized centers and university hospitals were viewed as valuable support structures, though still uncommon in Germany:

„It is always good to have a neutral specialist clinic around.“ (ID 41, specialist in dental fear treatment)

Education was seen as a critical factor. Nearly three quarters of respondents supported improved undergraduate training in managing anxious patients, though some favoured postgraduate education due to the need for prior clinical experience. Interest in continuing education was generally high, especially in formats offering practical approaches.
