# Supplementary material for: Perceived barriers, applied strategies, and typology of dentists treating patients with dental anxiety: a qualitative study
Source: BMC Oral Health. 2026 Feb 13;26:422. doi: 10.1186/s12903-026-07886-7 (PMC12955291; doi:10.1186/s12903-026-07886-7)
Supplement: Supplementary file 3 — Supplementary Material 3. [file 12903_2026_7886_MOESM3_ESM.docx]

## Appendix 3 – Detailed Description of the Four Dentist Types

In the main manuscript, the four types of dentists identified in this study are presented in a concise form to illustrate their key characteristics and implications. For readers who wish to gain a deeper understanding of these typologies, the following section provides more detailed descriptions based on recurring patterns observed during analysis. These profiles are derived from consistent response patterns across personality, prior knowledge, professional experience, and clinical approach.

**Type I – The Understanding Type with Minor Weaknesses (Understanders)**

Comprising 26.4% of the sample, this group perceives DA as highly relevant and has extensive experience with anxious patients. Many have expanded their knowledge through continuing education or literature. They generally feel capable of managing such patients and never entirely helpless, though remain somewhat uncertain about the best route to further improvement. Their approaches—ranging from refined communication techniques to the use of sedation or psychological support—demonstrate a strong willingness to reduce barriers. Motivation is evident, attitudes are predominantly positive and empathetic, and the first steps toward structured treatment concepts are visible.

**Type II – The Demanding Type with Self-Doubt (Demanders)**

Representing the largest group at 41.2%, Demanders are attuned to the needs of anxious patients, holding a largely neutral stance but still regarding them as a professional challenge. They recognize barriers such as time constraints, financial pressures, or personal insecurity and have adopted behavioural strategies to cope. Although rarely reaching a point of complete helplessness, they often experience stress and exertion. Nevertheless, they strive to maintain motivation through self-reflection and aim to emerge stronger from each encounter. They acknowledge the value of further training but also note that DA treatment is frequently unprofitable and that the range of relevant courses is limited.

**Type III – The Dissatisfied Improvisers (Improvisers)**

Accounting for 20.5% of respondents, Improvisers do not consistently differentiate their treatment of DA patients from that of other patients. They improvise when necessary but may feel overwhelmed when routines break down. Communication and treatment techniques can reach their limits, leading to frustration or uncertainty. Impatience and stress are common, particularly during complex or time-consuming treatments. While dissatisfied with their current situation, members of this group are often candid about their limitations—a trait that may signal openness to change if structured support were available.

**Type IV – The Practitioners without Guidance (Unguided)**

The smallest group at 11.3%, Unguided dentists frequently feel helpless or even desperate when treating anxious patients, which fosters sceptical or negative attitudes. Some describe being physically drained—“tired” or “sweating through their clothes”—after such treatments. They rarely adapt their approach and remain dissatisfied with the situation. Many doubt the practical value of university-level or continuing education in this area. Although they hope that experience will eventually lead to improvement, they lack a structured plan to address DA in their practice.
